# Association between Eating Habits and Sodium Intake among Chinese University Students

**DOI:** 10.3390/nu15071570

**Published:** 2023-03-24

**Authors:** Minchan Wu, Yue Xi, Jiaqi Huo, Caihong Xiang, Cuiting Yong, Jiajing Liang, Hanshuang Zou, Yunfeng Pan, Qingqing Xie, Qian Lin

**Affiliations:** 1 Department of Nutrition Science and Food Hygiene, Xiangya School of Public Health, Central South University, 110 Xiangya Rd, Changsha 410078, China; 2 Guangdong Provincial Key Laboratory of Food, Nutrition and Health, Department of Epidemiology, School of Public Health, Sun Yat-sen University, Guangzhou 510275, China

**Keywords:** dietary sodium intake, dietary habits, college students, eating out, takeaway

## Abstract

(1) Background: Insufficient evidence exists regarding the dietary habits that may contribute to high sodium intake among college students in China. This cross-sectional study aimed to investigate the dietary sodium intake of college students in Hunan and its association with their dietary habits. (2) Methods: In total, 585 university students from Hunan were recruited for this study. The sodium Food Frequency Questionnaire (sodium-FFQ) and dietary habits were assessed. (3) Results: Excluding cooking salt and high-sodium seasonings, the daily dietary sodium intake among college students in Changsha, Hunan Province, was 1183.74 (563.38, 2054.86) mg/day. A vast majority (89%) of college students reported eating outside of school at least once a week, and approximately one-third (34%) ordered takeaways at least once a week. After adjusting for confounding factors, the associations between the frequency of eating out and ordering takeaways with college students’ sodium intake remained significant. (4) Conclusions: The findings indicate that excessive dietary sodium intake among college students in Hunan is a growing concern. College students who frequently eat out and order takeaways tend to have a higher sodium intake. Future research should focus on identifying the main sources of dietary sodium and developing interventions that promote healthy dietary habits among college students.

## 1. Introduction

High dietary sodium intake is a well-known risk factor for hypertension, which, in turn, increases the risk of cardiovascular disease, stroke, and mortality [1,2,3,4]. The prevalence of excessive sodium intake is concerning, especially in China, as the majority of the population consumes more than the recommended amount [5,6,7]. Recent global surveys have shown an average daily sodium intake of 3950.0 mg/day (equivalent to 2 g/day salt) [6], with Chinese individuals having an average daily sodium intake of 4318.1 mg/day as of 2018 [8]. These values are significantly higher than the 2000 mg/day recommended by the World Health Organization (WHO) [9]. Therefore, how a reduction of dietary sodium intake can be achieved represents a crucial public health problem.

The identification of food sources that contribute to sodium intake is a critical step in reducing sodium consumption. Of particular concern is the sodium content hidden in processed foods and high-sodium seasonings, which is not easily detectable compared to sodium added through the addition of salt during cooking [10,11,12]. Previous research has shown that the majority of dietary sodium intake, up to 70%, is derived from processed foods [13,14]. This is especially concerning in China, where sodium intake from processed foods and Monosodium Glutamate (MSG) is on the rise [15]. Furthermore, individuals may not be aware of the amount of sodium they consume daily, as changes in sodium content in processed foods are not readily apparent [16].

It is worth noting that there have been notable changes in the structure and dietary habits of the younger generation [17], with a shift towards increased consumption of processed foods from outdoor sources such as restaurants, convenience stores, and takeaways [18]. A study showed that young people consumed approximately 40% of their total daily energy away from home in America [19]. Similar trends are observed in China, where approximately 27% of college students eat out every week in Chongqing [20] and 15.4% of young Chinese individuals order takeaway at least once a week [21]. Such dietary habits are generally considered less healthy than homemade food, with higher levels of energy, fat, salt, and sugar and lower levels of vitamins and minerals [22,23,24,25,26,27,28]. Therefore, this shift in dietary habits may lead to increased sodium intake among adolescents.

College students, typically defined as individuals between the ages of 18 and 25 years old, undergo a significant period of transition into adulthood, during which they experience remarkable changes in their dietary behavior and eating habits [28]. Upon entering college, students are exposed to a new living environment and lifestyle [28] and are granted autonomy in their food choices [29], which, coupled with the ease of access provided by the internet [30], may lead to shifts in their dietary patterns. As a large and influential demographic, the dietary habits of Chinese college students have implications not only for their own health in adulthood, but also for the health of their social network and society at large [31]. Hunan Province, in China, is experiencing a rapid economic development, leading to a complex food environment that can greatly impact the eating habits of college students. Despite this, there is a lack of comprehensive research on the dietary patterns of college students in Hunan province. Therefore, gaining insights into the dietary patterns of Hunan university students and examining the association with sodium intake is crucial to enhancing the nutritional status of the population.

Our study was conducted at three Universities in Hunan province, with a diverse student body, of approximately 25,000 students enrolled across various disciplines. The majority of students are of Han ethnicity, with a small percentage of students from other ethnic groups. The university attracts students from various socioeconomic backgrounds, both from urban and rural areas within Hunan Province and other regions in China.

The objective of this study was to examine the relationship between dietary sodium intake and poor dietary habits among Chinese university students in Hunan. To achieve this goal, we employed the sodium Food Frequency Questionnaire (sodium-FFQ), which was adjusted by the 24 h urinary sodium, to quantify the dietary sodium intake of university students. Our hypothesis was that poor dietary habits would increase the likelihood of high sodium intake among college students. By shedding light on the relationship between dietary habits and sodium intake, this study has the potential to inform health promotion strategies for Chinese college students by promoting the control of poor dietary habits and reducing sodium intake.

## 2. Materials and Methods

### 2.1. Ethical Approval

This study received consent from the Ethics Review Committee of the Xiangya School of Public Health, Central South University (XYGW-2020-087).

### 2.2. Study Design and Participants

This cross-sectional study was conducted in Changsha city, located in Hunan Province, China, from September to December 2021. Participants were eligible if they met the following criteria: (1) absence of serious diseases such as liver and kidney failure, cardiovascular and cerebrovascular diseases, benign tumors, or mental illnesses; (2) regular eating habits in the past month without being on a diet; (3) ability to read and write; and (4) enrollment in the universities located in Hunan province. Eligible college students were recruited using convenient sampling methods. Based on a previous study [32], the mean 24-hour urine sodium intake of adult residents in Changsha was approximately 3290.20 mg, with a standard deviation of 705.88 mg/day. The allowable error was set at 10% of the standard deviation, which equates to 0.07 mg/day. Using the formula for estimating sample size based on the overall mean, a sample size of 385 was calculated.

(1) $$n={\left(\frac{u_{\alpha /2}\sigma }{\delta }\right)}^{2}$$

To account for potential dropouts or incomplete data, we aimed to recruit 585 participants.

### 2.3. Data Collection

We employed two stages of stratified sampling to ensure that our sample was representative of the overall student population in terms of age, gender, and academic discipline. We contacted teachers in different departments and asked them to help recruit students participating in our study. We also advertised the study through the university’s social media channels and posted flyers around the campus. After understanding the study’s objective and eligibility requirements, interested students could scan the QR code to read the electronic informed consent form. Once they provided their consent, they completed an online questionnaire using Questionnaire Star (www.wjx.cn accessed on 4 March 2021). Participation in the study was completely voluntary, and students were at liberty to discontinue their involvement at any point. The questionnaire collected demographic information and knowledge, attitudes, and practices (KAP) data on salt, food environment, dietary habits, and dietary sodium intake.

#### 2.3.1. Demographic Characteristics

College students’ age, sex, ethnicity, college, major, grade, and pocket money per month were collected using a self-administered questionnaire. We also collected data on students’ self-reported height and weight and then calculated body mass index (BMI, kg/m^2^). Following the BMI categorization of Chinese adults, the BMI range of healthy adults is 18.5~23.9 kg/m^2^, with those under the range defined as underweight. Those whose BMI is between approximately 24.0 and 27.9 kg/m^2^ are overweight and those above 28 kg/m^2^ are obese.

#### 2.3.2. Knowledge, Attitudes, and Practices about Salt

We administered a KAP questionnaire to evaluate participants’ knowledge, attitudes, and practices related to salt, which was adapted from a previous study [33]. The knowledge section consisted of five questions related to salt, such as recommended daily salt intake, the association between salt and hypertension, the link between salt intake and strength, awareness of low sodium salt, and identification of the symbol for salt content on the food composition table. Each question was scored as 1 for a correct response, 0 for an incorrect answer, or “don’t know,” and the maximum total score was 5. A score ≥ 3 was considered indicative of a high level of salt-related knowledge. The attitudes section included two questions regarding willingness to reduce salt consumption in daily life. A score of 1 was awarded for selecting “yes,” while 0 was given for “no” or “don’t know,” and the maximum total score was 2. A score ≥ 1 was deemed as having a positive attitude related to salt. The practices section included five questions designed to assess unfavorable salt-related behaviors, such as “do you still try to eat food if it is very salty?” A score of 0 points was assigned for choosing a behavior, and 1 was assigned for not selecting it. The maximum total score was 5. A score ≥ 3 was defined as having good behavior related to salt.

#### 2.3.3. Food Environment

The modified food environment perception questionnaire [34] is used to gauge college students’ self-perception of their food environment. The community food environment questionnaire was validated (Cronbach’s α = 0.822). This questionnaire has 18 questions corresponding to 3 dimensions. For food availability and food purchasability, each question was answered based on a 4-point option expressing agreement (4 = “very agree,” 3 = “agree,” 2 = “neutral,” 1 = “disagree”/“completely disagree), and price (4 = “very cheap,” 3 = “cheap, 2 = “reasonable, 1 = “expensive”/“very expensive”). Food accessibility had four options (4 = “less than or equal to 10 min”, 3 = “11~20 min”, 2 = “21~30 min”, and 1 = “greater than 30 min”). Higher scores for each dimension indicated that residents have more adequate, cheaper, and convenient access to abundant food. Levels were classified as high or low according to the median grouping of each dimension score.

#### 2.3.4. Eating Habits

Participants were administered a self-reported questionnaire to elicit information on their dietary habits.

(1) Main dietary behavior patterns: “Where do you usually go to eat most often?” This was a single-choice question, in which the answers were divided into student canteen, restaurant (excluding takeaway), home (including rented-out homes), ordering takeaways, or “other way,” which allowed for free-text input.

(2) Questions about outside eating: In this study, participants were surveyed regarding their eating habits outside of the campus over the course of the preceding month. Respondents who indicated that they had eaten outside of the campus during that time were then asked to specify the frequency of their dining-out experiences on a weekly basis. Responses were classified into five categories: “1~2 times,” “3~5 times,” “6~10 times,” “more than 10 times,” and “don’t remember.” Students were then divided into two groups based on the frequency of their dining-out experiences: “<1 time a week” and “≥1 time a week” [35,36]. Responses of “don’t remember” were excluded from the subsequent analysis. For students who had eaten outside of the campus, information was collected regarding their preferred restaurants, which were defined as those visited four or more times per month, and subsequently categorized based on prior studies [20,37], as presented in Table 1.

(3) Takeaway question: In this study, participants were queried regarding their consumption of takeaway meals within the past month. Those who answered in the affirmative were subsequently asked to specify the frequency of their dining-out experiences on a weekly basis. Responses were categorized into five groups: “more than 14 times,” “7~14 times,” “4~6 times,” “less than or equal to 3 times,” and “don’t remember.” Participants were then sorted into two groups, “<1 time a week” or “≥1 time a week,” with the latter group being considered “frequent takeaway order.” Notably, participants who responded with “don’t remember” were excluded from further analysis. Additionally, information on participants preferred takeaway establishments, defined as those visited four or more times per month, was collected and classified according to Table 1.

#### 2.3.5. Assessment of Sodium Intakes

Dietary sodium consumption of undergraduate students was accessed by the modified sodium Food Frequency Questionnaire (modified sodium-FFQ). This sodium-FFQ has been validated, and its Spearman correlation coefficient of test–retest between the Sodium-FFQ and 3 × 24 h dietary record was 0.393 (*p <* 0.05). Furthermore, the sodium-FFQ was related to 24 h urinary sodium-to-potassium ratio by their Spearman coefficient of 0.370 (*p <* 0.05). The modified sodium-FFQ includes 68 items, and all items are set in an open format, leading to college students filling them in on their own. The consumption frequency of each item, answered by ‘time(s)/month,’ ‘time(s)/week,’ and ‘time(s)/day,’ was divided by 28 days (a mouth) to calculate the daily frequency. The total sodium intake was estimated using the formula as follows:

(2) $$Total\;Na=\sum_{i=0}^{n}F_{i}\times I_{i}\times C_{Na,i}$$

Total sodium intake from FFQ (total Na, mg/day), whether to add salt again or not (AS, yes *=* 1, no *=* 0), frequency of adding salt (FAS, occasionally *=* 1/7 *=* 0.1429, often *=* 3/7 *=* 0.3571), grams of salt added each time (mAS, one spoonful *=* 2 g, unclear *=* 3 g, or the actual amount filled by the subjects), and the conversion factor (1 g salt ≈ 400 mg sodium). The median dietary sodium intake of college students was calculated and those above the median were defined as “high sodium intake”.

### 2.4. Statistical Analyses

Data cleaning and analyses were conducted using SPSS 26.0 software (IBM Corp., Armonk, NY, USA). Moreover, the figures were visualized using GraphPad Prism 8.0.1 (GraphPad Software, Inc., San Diego, CA, USA). Chi-squared test and student *t*-test were used to identify demographic data for categorical or continuous variables. Binary logistic regression analyses were employed to assess the association between eating habits and dietary sodium intake. The explanatory variables with *p <* 0.10 were included in the multivariate analyses by the binary logistic regression’s backward stepwise method. The significance level was set at *p <* 0.05.

## 3. Results

### 3.1. Characteristics of College Students in Terms of Sodium Intakes and Dietary Habits

Of the 654 students contacted, 585 completed the survey, yielding a response rate of 89.4%. The final sample consisted of 325 females and 260 males, with an average age of 19.1 years (Table 2). The participants represented a diverse range of academic disciplines, closely reflecting the overall student population at Universities in Hunan. The dietary sodium intake of Hunan college students was 1183.74 mg/day (563.38, 2054.86) mg/day, and dietary sodium intake higher than 1183.74 mg/day was defined as “high sodium intake”. Among them, 44.4% of the participants were male, and the most-represented ethnicity was Han (89.6%). The college students came from various universities in Hunan, mainly Central South University (63.8%) and Hunan University of Traditional Chinese Medicine (20.0%). Nearly half of the participants (42.2%) majored in medicine, and about the half students (51.8%) were college freshmen, followed by college sophomores (18.5%). More than half of the students’ pocket money (58.3%) was less than RMB 1500 monthly. For the participants’ self-reported BMI status, the vast majority (68.4%) were of normal body type, while 14.7% were overweight or obese. The proportion of college students with high scores in salt-related knowledge, attitudes, and behaviors was decreasing in order (73.5% vs. 59.3% vs. 12.8%). Females had a higher likelihood of consuming more dietary sodium than males (44.6% vs. 55.1%, *p =* 0.012). Males, on the other hand, reported eating out more frequently than females (86.8% vs. 51.3%, *p =* 0.041). Additionally, females significantly preferred spicy snacks compared to males (43.1% vs. 62.8%, *p =* 0.000, Table S1). College students with greater availability to food were more likely to consume a high dietary sodium intake (46.7% vs. 54.6%, *p =* 0.049).

The majority of college students (89.7%) ate out more than once a week, and nearly half of the students (30.9%) ordered takeaways more than once a week (Table 2). Males ate out more frequently than females (86.8% vs. 51.3%, *p =* 0.041, Table S1). Moreover, monthly pocket money was correlated with the frequency of dining out (*p =* 0.002) and ordering takeaways (*p <* 0.001). Compared to non-medical students, medical students ordered takeaways more frequently (37.4% vs. 26.2%, *p =* 0.004), and had a higher level of salt-related knowledge (81.8% vs. 67.5%, *p* < 0.001, Table S2). It was also found that students with greater food accessibility were more likely to eat out frequently (87.3% vs. 92.6%, *p =* 0.040).

### 3.2. Sodium Intake and Eating Habits among College Students

In the current study, the most common method of consuming meals among college students was found to be in the student canteen (76.8%), followed by ordering takeaway food (12.0%) (Table 3). No significant difference was observed between the major types of eating and the levels of sodium intake (*p =* 0.547). However, students who ate out more frequently had a higher likelihood of having a high sodium intake (47.7% vs. 52.3%, *p =* 0.010). Furthermore, a positive correlation was found between the frequency of takeaway ordering and higher sodium intake (37.9% vs. 67.1, *p <* 0.001). Only a small proportion of students (8.4%) reported adding salt to their cooked meals. More than half of the participants (54.0%) reported consuming spicy food daily. A difference between adding salt to a cooked meal (*p =* 0.268), liking spicy snacks (*p =* 0.823), and sodium intake levels warrants further investigation.

### 3.3. Type of Favorite Restaurants for College Students to Go out or Order Takeaway

**Figure 1:**
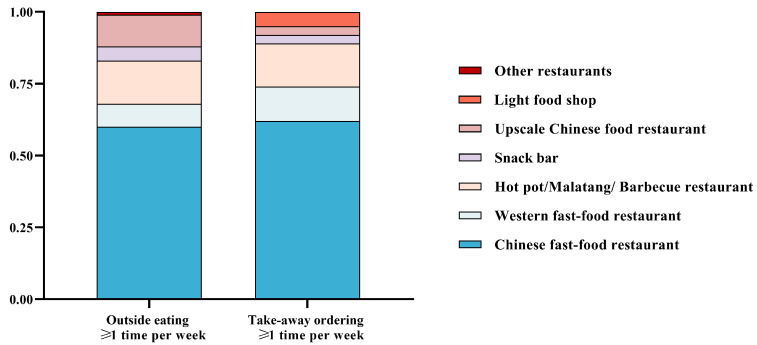
Type of favorite restaurants for college students in Hunan who were outside eating ≥1 time pre-week or takeaway ordering ≥1 time pre-week.

The results of this study indicate that the majority of Hunan college students (59.53%) prefer Chinese fast food as their top choice when eating out, followed by hot pot/malatang/barbecue (11.7%) and upscale Chinese food restaurants (11.03%) (Figure 1). Furthermore, Chinese fast-food restaurants are also the most popular choice for ordering takeaway among the participants, accounting for 62.00% of all orders, with Western fast-food restaurants coming in second place (12.10%). Unlike eating out, students still considered light food shops when ordering takeaways (0.00% vs. 0.05%). In summary, the findings suggest that Chinese fast-food restaurants are the most commonly selected food establishments by college students in Hunan, both for eating out and ordering takeaways.

### 3.4. The Association between Eating Habits and Sodium Intake among College Students

The logistic regression analysis (Table 4) in this study revealed that female college students had a higher likelihood of consuming higher levels of dietary sodium compared to male students, even after adjusting for other variables (adjusted OR = 1.457, 95% CI: 1.029~2.064, *p* < 0.05). In addition, college students who eat out more often are at higher risk of consuming higher levels of dietary sodium (crude OR = 2.179, 95% CI: 1.097~3.588, *p* < 0.001). This situation remains after adjusting for variables including age and gender (adjusted OR = 1.983, 95% CI: 1.097~3.588, *p* < 0.05). Similarly, students who ordered takeaways once a week or more had a higher likelihood of consuming more sodium, even after adjusting for sex, age, pocket money, self-reported BMI, and food availability (adjusted OR = 2.027, 95% CI: 1.390~2.957, *p* < 0.001).

## 4. Discussion

The aim of this investigation was to evaluate the correlation between dietary sodium consumption and dietary patterns among university students in China. Our findings indicate that Chinese university students already consume over 50% of the recommended daily sodium intake (2400 mg/day), as per the 2018 Chinese guidelines for hypertension management [38], though their diet, without considering salt and most high-sodium condiments. A vast majority of the students frequently dine out off-campus at least once a week, with almost half of them choosing to order takeaway meals once a week. It is noteworthy that a higher frequency of eating out and ordering takeaways is directly associated with increased sodium intake from meals. Additionally, it is important to note that the most commonly preferred dining options for Hunan university students are Chinese fast-food restaurants.

The present study revealed that the median dietary sodium intake of college students in Hunan was 1183.74 mg/day, which is equivalent to consuming 3 g of salt per day. This suggested that college students in Hunan were already consuming approximately 60% of the recommended daily sodium intake from both processed and natural foods alone. Unfortunately, the largest portion of Chinese dietary sodium is added to the diet as seasoning salt [39,40,41]. Previous studies have found that the median sodium content in Chinese restaurant dishes is 487 mg per 100 g, with 74.9% of dishes containing over 1500 mg of sodium per serving [42]. Thus, the excessive dietary sodium intake of Chinese college students is a remarkably important public health issue.

In this study, we discovered that dietary sodium intake was higher in females than in males, which was different from the results of some other studies [43,44,45]. This is potentially because this study focused on sodium in processed and natural foods and did not take into account seasoning and salt addition. Meanwhile, the dietary preferences brought about by gender make females prefer processed foods and snack foods compared to males [46,47], which can lead to higher sodium intake. While inconsistent with other studies, a relationship between BMI and sodium intake was not discovered, probably because of information bias in self-reported BMI by students as the primary reason [48,49].

In comparison with prior research [50,51], our study indicated that behavior was the only salt-related KAP associated with dietary sodium intake among college students. Additionally, based on our findings, college students scored the highest in knowledge related to salt intake, followed by a decrease in the proportion of high scores in attitudes, and the lowest high scores in behavior. These results emphasize the importance of addressing behavioral feasibility when educating college students on sodium intake. Therefore, future nutrition education interventions should focus on improving the feasibility of healthy behaviors related to sodium intake. Moreover, this study provides novel insights into the impact of food environment on dietary sodium intake among college students. Specifically, food availability was found to be significantly associated with higher sodium intake, whereas food accessibility did not demonstrate a significant association. The higher food availability could lead to increased food consumption and subsequently, greater sodium intake. Furthermore, the advent of take-out has made food more accessible, which may have compensated for the physical distance between college students and food sources. These findings underscore the importance of considering the impact of food environment, specifically food availability, on dietary sodium intake among college students.

Compared to primary and secondary school students, college students enjoy greater autonomy in selecting their meal venues, which includes on-campus cafeterias, as well as off-campus eateries and takeout options. In Hunan, over 80% of college students surveyed reported dining outside of the school cafeteria at least once per week, with nearly half opting for takeout. These findings align with similar studies conducted by Fan [52] and Adams [53]. Among Chinese college students, dining out and takeaway are becoming more and more popular due to the popularity of electronic delivery platforms, the rapid increase in delivery efficiency, convenience, and low price [54]. Moreover, college students are at risk for depression, anxiety, and other mental problems [55,56]. They face significant various forms of pressure, including academic [57], social pressure [58], employment pressure [45], and even debt pressure [59,60]. Consequently, these pressures may lead to emotional eating and a preference for energy-dense foods [61,62], ultimately prompting college students to forego on-campus dining options in favor of dining out or ordering takeout.

Our study further confirms the positive association between eating out and high dietary sodium intake among college students. This discovery is consistent with a cross-sectional study conducted by Zang in Shanghai which found eating at restaurants was associated with an increased intake of 548 mg of sodium [63]. Further, Llanaj found that eating out can lead to higher dietary sodium intake [64]. Compared to school can-teens, restaurants tend to serve unhealthier food with higher levels of oil, sugar, and salt [19,54]. To enhance the taste, texture, color, and flavor of food, restaurants often add excessive amounts of salt, sugar, and oil and employ unhealthy cooking methods, such as deep-frying and stir-frying [63]. When college students opt for more appealing foods in restaurants instead of the cafeteria, they inevitably consume higher amounts of sodium.

Despite the comprehensive evaluation of dietary sodium intake among Chinese college students, the current study did not find an association between the habit of adding salt to meals and sodium intake due to a limited number of individuals who reported this behavior [50,51,52]. The decision to add salt to meals is typically driven by personal preference [65] and habitual salt intake [66], and it is more prevalent in Western countries [67]. It is also noteworthy that Chinese college students tend not to carry salt with them since they have limited opportunities to cook for themselves, which further decreases the likelihood of repeated salt addition during meals. Future studies may need to explore this behavior in a more diverse sample to further understand its impact on sodium intake among college students.

In China, spices are considered to be fundamental taste components [68]. A recent study revealed that a substantial proportion, over 30%, of Chinese adults consume spicy foods on a daily basis [69]. Interestingly, a positive association between the intake of spicy foods and preference for salty flavors has been reported [70], possibly due to the fact that spicy Chinese snacks often contain excessive amounts of oil and spices. This may be attributed to the infrequent consumption of spicy snacks by college students. Future investigations are warranted to examine the impact of the frequency of spicy snack intake on dietary sodium intake among university students in Hunan province.

Nearly half of the study population comprised medical students, who exhibited better knowledge of salt intake compared to non-medical students. However, no significant difference in dietary sodium intake was observed between medical and non-medical students. Additionally, medical students were found to order takeaway more frequently than non-medical students, possibly due to their stressful academic schedules leading to fatigue and unhealthy consumption behaviors [71,72,73,74]. The findings highlight the need for targeted interventions to promote healthy eating habits among medical students, who are an important subgroup of the college population.

Our study demonstrated several noteworthy strengths. We employed a validated sodium-FFQ specifically designed for Chinese university students to comprehensively estimate dietary sodium intake, thereby producing credible data on sodium intake. The sodium-FFQ includes processed foods high in sodium as well as Hunan special foods, enabling a more accurate evaluation of salt in processed foods and snacks. To the best of the authors’ knowledge, no comparable study has employed this sodium-FFQ in China. Moreover, the KAP-related salt and community food environment instruments used in the study have undergone testing for reliability and validity. Additionally, the participants were required to complete the questionnaire under the supervision of teachers and assistants to ensure data integrity.

Despite these strengths, several limitations exist in our study. Firstly, as a cross-sectional study, we cannot establish a causal relationship between dietary habits and dietary sodium intake. Secondly, our study has achieved the predetermined sample size required to accurately represent the Hunan University student population. However, it is important to note that the study was conducted among college students residing in a provincial capital city in Central China, which may limit the generalizability of the findings to the wider population of Chinese college students. Moreover, the self-reported questionnaires used to assess sodium intake may have resulted in some information bias. Lastly, we did not measure the blood pressure levels of the students, which prevented us from further exploring the relationship between dietary habits, dietary sodium intake, and blood pressure.

## 5. Conclusions

The high prevalence of eating out and ordering takeaways among Chinese college students is concerning, as it is associated with higher dietary sodium intake. Specifically, students who frequent Chinese fast-food restaurants are at risk for consuming unhealthy levels of sodium. As such, there is a need for sodium-related nutrition education targeted at college students, especially those with unhealthy eating habits. Additionally, restaurants and take-out stores could play a role in providing information about the sodium content of their food offerings, such as through menu labeling. Future research should focus on identifying specific food sources of dietary sodium among college students and developing effective interventions to promote healthier eating habits.

## Figures and Tables

**Table 1:** The classification of food stores.

Classification	Definition
Chinese fast-food restaurant	Chain or individually managed Chinese restaurants offer a limited range of Chinese cuisine, including fried rice, noodles, rice, and much more.
Western fast-food restaurant	Restaurants mainly provide hamburgers, pizza, fried chicken, and much more, including KFC, and McDonalds.
Upscale Chinese food restaurant	The restaurants offer diversified Chinese diets as well as a la carte service, with seating for over 75.
Hot pot/Malatang/barbecue restaurant	The restaurants offer spicy dishes such as a hot pot or spicy hotpot or food baked with special seasonings.
Snack Bar	The stores specialize in pickled fruits, cookies, pastries, and beverage much more.
Light Food restaurant	The restaurants mainly sell low-salt, fat, and oil-light meal sets.
Other restaurants	The restaurants offer foods, not from China, such as Korean and Japanese foods.

**Table 2:** Characteristics of participants by sodium intake and frequency of eating out, and ordering takeaway (n *=* 585, % or mean ± SD).

Variables	Total 585 (100.0)	High Sodium Intake 295 (50.4)	*p*	Outside Eating #	*p*	Takeaway Ordering #	*p*
				≥1 Time per Week		≥1 Time per Week	
				507 (89.7)		174 (30.9)	
Age	19.06 ± 1.45	19.13 ± 1.36	0.863	19.05 ± 1.45	0.151	19.20 ± 1.42	0.505
Sex			**0.012**		**0.041**		0.148
Male	260 (44.4)	116 (44.6)		217 (86.8)		70 (27.8)	
Female	325 (55.6)	179 (55.1)		290 (51.3)		104 (33.4)	
Ethic			0.381		0.980		0.314
Han	524 (89.6)	261 (49.8)		454 (89.7)		160 (31.6)	
other	61 (10.4)	34 (55.7)		53 (89.9)		14 (25.0)	
Major			0.682		0.067		**0.004**
Medicine	247 (42.2)	127 (51.4)		221 (92.5)		89 (37.4)	
Other	338 (57.8)	168 (49.7)		286 (87.7)		85 (26.2)	
Grade			0.737		0.453		**0.038**
Freshman	303 (51.8)	146 (47.6)		266 (90.8)		80 (27.4)	
Sophomore	108 (18.5)	60 (52.8)		95 (91.3)		37 (36.3)	
Junior	106 (18.1)	56 (52.8)		89 (85.6)		28 (27.5)	
Senior year and above	68 (11.6)	36 (50.4)		57 (89.1)		29 (43.3)	
Pocket money			0.419		**0.002**		**<0.001**
≤1000 RMB	107 (18.3)	56 (52.3)		87 (84.5)		29 (28.7)	
1001~1500 RMB	234 (40.0)	111 (47.4)		192 (86.1)		41 (18.2)	
1501~2000 RMB	173 (29.6)	95 (54.9)		164 (96.5)		67 (40.1)	
>2000 RMB	71 (12.1)	33 (46.5)		64 (92.8)		37 (52.9)	
Self-reported BMI (kg/m^2^) *			0.743		0.780		0.195
Underweight	97 (16.6)	51 (52.6)		86 (90.5)		28 (29.5)	
Normal	402 (68.4)	198 (49.3)		348 (90.2)		125 (32.6)	
Overweight	75 (12.8)	39 (52.0)		63 (86.3)		16 (21.6)	
Obesity	11 (1.9)	7 (63.6)		10 (90.9)		5 (45.5)	
Salt-Related Knowledge, Attitude, and Behaviors Status							
Salt-related knowledge			0.976		0.550		**0.041**
Low	155 (26.5)	78 (50.3)		132 (91.0)		35 (24.1)	
High	430 (73.5)	217 (50.5)		375 (89.3)		139 (33.3)	
Salt-related attitude			0.864		0.674		0.203
Low	238 (40.7)	119 (50.0)		204 (89.1)		77 (33.9)	
High	347 (59.3)	176 (50.7)		303 (90.2)		97 (28.9)	
Salt-related behaviors			**0.015**		**<0.001**		**<0.001**
Low	510 (87.2)	267 (53.2)		466 (94.7)		168 (34.4)	
High	75 (12.8)	28 (37.3)		41 (56.2)		6 (8.1)	
Self-perceived food environmental settings							
Food Availability			**0.049**		**0.040**		0.688
Low	321 (54.9)	150 (46.7)		269 (87.3)		98 (31.6)	
High	264 (45.1)	145 (54.6)		238 (92.6)		76 (43.7)	
Food Accessibility			0.496		0.496		0.389
Low	412 (70.4)	204 (49.5)		354 (89.2)		127 (32.0)	
High	173 (29.6)	91 (52.6)		153 (91.1)		47 (28.3)	
Food Purchasability			0.730		0.550		0.511
Low	377 (57.6)	172 (51.0)		294 (89.1)		104 (32.0)	
High	248 (42.4)	123 (49.6)		213 (90.6)		70 (29.4)	

Compared by the Chi-square test and student *t*-test. * BMI: body mass index. # Data with missing values. RMB: Renminbi, Chinese official coupons, 1 RMB ≈ 0.14 USD. Universities have set up five-year programs in some majors in China. Bolding indicates statistically significant values, *p* < 0.05.

**Table 3:** Dietary habits associated with sodium intakes among college students.

Variables	Total 585 (100.0)	High Sodium Intake	x ^2^	*p*
Main ways of dietary behavior			2.125	0.547
Students’ canteen	456 (76.8)	221 (48.5)		
Takeaway food	71 (12.0)	41 (57.7)		
Restaurant	57 (9.6)	28 (49.1)		
Home	10 (1.7)	5 (50.0)		
Outside eating #			6.586	**0.010**
<1 time per week	58 (10.3)	20 (34.5)		
≥1 time per week	507 (89.7)	151 (52.3)		
Takeaway ordering #			13.202	**<0.001**
<1 time per week	389 (69.1)	177 (45.5)		
≥1 time per week	117 (30.9)	108 (62.1)		
Re-adding salt to cooked meals			1.226	0.268
Yes	49 (8.4)	21 (42.9)		
No	536 (91.6)	526(91.6)		
Like spicy snacks			0.050	0.823
Yes	316 (54.0)	158 (50.0)		
No	269 (46.0)	137 (50.9)		

Compared by Chi-square test. # Data with missing values. Bold indicates statistically significant values, *p* < 0.05.

**Table 4:** Binary logistic regression models of the association between dietary habits and sodium intakes among college students (n = 585).

Variables	Crude OR (95% CI)	Adjusted OR (95% CI)
Sex (male = *Ref*)	1.438 (1.017, 2033) *	1.457 (1.029, 2.064) *
Outside eating (<1 time per week = *Ref*)	2.179 (1.097, 3.588) ***	1.983 (1.097, 3.588) *
Takeaway ordering (<1 time per week = *Ref*)	2.031 (1.397, 2.952) ***	2.027 (1.390, 2.957) ***

*** *p* < 0.001 and * *p* < 0.05. OR, odds ratio. 95% CI, 95% confidence interval. Adjusted by pocket money, self-reported BMI, food availability, and salt-related behaviors.

## Data Availability

The data that support the findings of this study are not publicly available due to the data containing information that could compromise participants’ privacy but are available from the corresponding author on reasonable request.

## Supplementary Materials

The following supporting information can be downloaded at: https://www.mdpi.com/article/10.3390/nu15071570/s1, Table S1: Characteristics of participants by re-adding salt to cooked meals and liking spicy; Table S2: Characteristics of participants by Salt-Related Knowledge, Attitude, and Behaviors Status.
