# Supplementary material for: Association between Eating Habits and Sodium Intake among Chinese University Students
Source: Nutrients. 2023 Mar 24;15(7):1570. doi: 10.3390/nu15071570 (PMC10097125; doi:10.3390/nu15071570)
Supplement: Supplementary file 1 [file nutrients-15-01570-s001.zip › nutrients-2279865-supplementary.pdf]

Table S1. Characteristics of participants by re-adding salt to cooked meals and liking spicy snacks. (n = 585, % or mean ± SD)

Variables	Total<br>585 (100.0)	Re-adding salt to cooked meals<br>Yes	*P*	Like spicy snacks<br>Yes	*P*
Age	19.06±1.45				
Sex			0.714		**0.000**
Male	260 (44.4)	23 (8.8)		112 (43.1)	
Female	325 (55.6)	26 (8.0)		204 (62.8)	
Ethic			0.303		0.408
Han	524 (89.6)	46 (8.8)		280 (53.4)	
Other	61 (10.4)	3 (4.9)		36 (59.0)	
Major			0.086		0.442
Medicine	247 (42.2)	15 (6.1)		138 (55.9)	
Other	338 (57.8)	34 (10.1)		178 (52.7)	
Grade			0.554		0.136
Freshman	303 (51.8)	29 (9.6)		169 (55.8)	
Sophomore	108 (18.5)	8 (7.4)		52 (48.1)	
Junior	106 (18.1)	9 (8.5)		64 (60.4)	
Senior year and above	68 (11.6)	3 (4.4)		31 (45.6)	
Pocket money			**0.009**		0.180
≤ 1, 000 RMB	107 (18.3)	15 (14.0)		56 (52.3)	
1, 001~1, 500 RMB	234 (40.0)	14 (10.3)		139 (59.4)	
1, 501~2, 000 RMB	173 (29.6)	8 (4.6)		87 (50.3)	
> 2, 000 RMB	71 (12.1)	2 (2.8)		34 (47.9)	
Self-reported BMI (kg/m2)*			0.444		0.922
Underweight	97 (16.6)	10 (10.3)		53 (54.6)	
Normal	402 (68.4)	34 (8.5)		215 (53.5)	
Overweight	75 (12.8)	5 (6.7)		41 (54.7)	
Obesity	11 (1.9)	0 (0.0)		7 (63.6)	
Salt-Related Knowledge, Attitude, and Behaviors Status					
Salt-related knowledge			**0.042**		0.528
Low	367 (62.7)	19 (12.3)		87 (56.1)	
High	218 (37.3)	30 (7.0)		229 (53.3)	
Salt-related attitude			**< 0.001**		**0.001**
Low	238 (40.7)	32 (13.4)		148 (62.2)	
High	347 (59.3)	17 (4.9)		168 (48.4)	
Salt-related behaviors			**0.003**		0.383
Low	344 (58.8)	36 (7.1)		279 (54.7)	
High	241 (41.2)	13 (17.3)		37 (49.3)	
Self-perceived food environmental settings					
Food Availability			0.125		**0.023**

Low	321 (54.9)	32 (10.0)		187 (58.3)	
High	264 (45.1)	17 (6.4)		129 (48.9)	
Food Accessibility			0.251		0.778
Low	412 (70.4)	31 (7.5)		221 (53.6)	
High	173 (29.6)	18 (10.4)		95 (54.9)	
Food Purchasability			0.711		0.872
Low	377 (57.6)	27 (8.0)		183 (54.3)	
High	248 (42.4)	22 (8.9)		133 (53.6)	

Compared by chi-square test. # compared by Fisher's precision probability test.

Table S2. Characteristics of participants by Salt-Related Knowledge, Attitude, and Behaviors Status. (n = 585, % or mean ± SD)

**Variables**	**Total 585 (100.0)**	**Salt-related knowledge High**	***P***	**Salt-related attitude High**	***P***	**Salt-related behaviors High**	***P***
Sex			0.557		0.292		**< 0.001**
Male	260 (44.4)	188 (72.3)		148 (56.9)		49 (44.4)	
Female	325 (55.6)	242 (74.5)		199 (61.2)		325 (55.6)	
Major			**< 0.001**		0.202		0.442
Medicine	247 (42.2)	202 (81.8)		154 (62.3)		138 (55.9)	
Other	338 (57.8)	228 (67.5)		193 (57.1)		178 (52.7)	
Grade			0.166		0.275		0.220
Freshman	303 (51.8)	214 (51.8)		46 (38.8)		173 (57.1)	
Sophomore	108 (18.5)	108 (18.5)		9 (8.3)		62 (57.4)	
Junior	106 (18.1)	106 (18.1)		13 (12.3)		64 (62.9)	
Senior year and above	68 (11.6)	68 (11.6)		7 (8.7)		48 (40.3)	
